# Transfemoral transcatheter aortic valve implantation in a patient with multiple endovascular aortic stents – a case report

**DOI:** 10.1186/s13019-016-0421-3

**Published:** 2016-02-02

**Authors:** Carolyn Weber, Antje-Christin Deppe, Kaveh Eghbalzadeh, Maximilian Scherner, Daphne Gray, Payman Majd, Michael Gawenda, Stephan Rosenkranz, Tanja Rudolph, Navid Madershahian, Thorsten Wahlers

**Affiliations:** Department of Cardiothoracic Surgery, University of Cologne, Kerpener Strasse 62, 50937 Cologne, Germany; Department of Vascular Surgery, University of Cologne, Cologne, Germany; Department of Cardiology, University of Cologne, Cologne, Germany

**Keywords:** TAVI, TF TAVI, Infra-renal aneurysm, Endovascular stents

## Abstract

**Background:**

In patients undergoing transfemoral transcatheter aortic valve implantation, previous endovascular interventions bear a risk for the valve frame to get stucked to the aortic stents.

**Case Presentation:**

We report on a 75-year-old frail patient with severe aortic stenosis and a rapid increase of an infra-renal aneurysm. He had a history of multiple endovascular interventions on the aorta. Due to his frail preoperative status we decided to perform a transfemoral transcatheter aortic valve implantation in combination with a simultaneous surgical abdominal aneurysm repair. To allow an atraumatic passage of the Edwards SAPIEN 3 valve across the endovascular stents we used a special technique.

**Conclusions:**

The transfemoral approach in patients with previous endovascular stenting can be performed successfully by a partial inflation of the distal balloon.

## Background

In patients precluded for conventional aortic valve replacement due to advanced age and multiple comorbidities, transcatheter aortic valve implantation (TAVI) is the standard of care [1]. Regarding the transfemoral (TF) approach there have been concerns in patients with aorto-iliac aneurysms, occlusive disease and excessive vessel tortuosity, which may complicate a safe TF access [2]. Some centers avoid TF TAVI in patients with aorto-iliac disease or aortic aneurysms due to exclusion of these patients from PARTNER trial and the risk of vascular complications [1]. Furthermore previous endovascular interventions bear a risk for the valve frame to get stucked to the aortic stents. Published experience of techniques to establish adequate TF access in these patients is limited.

## Case presentation

**Fig. 1:**
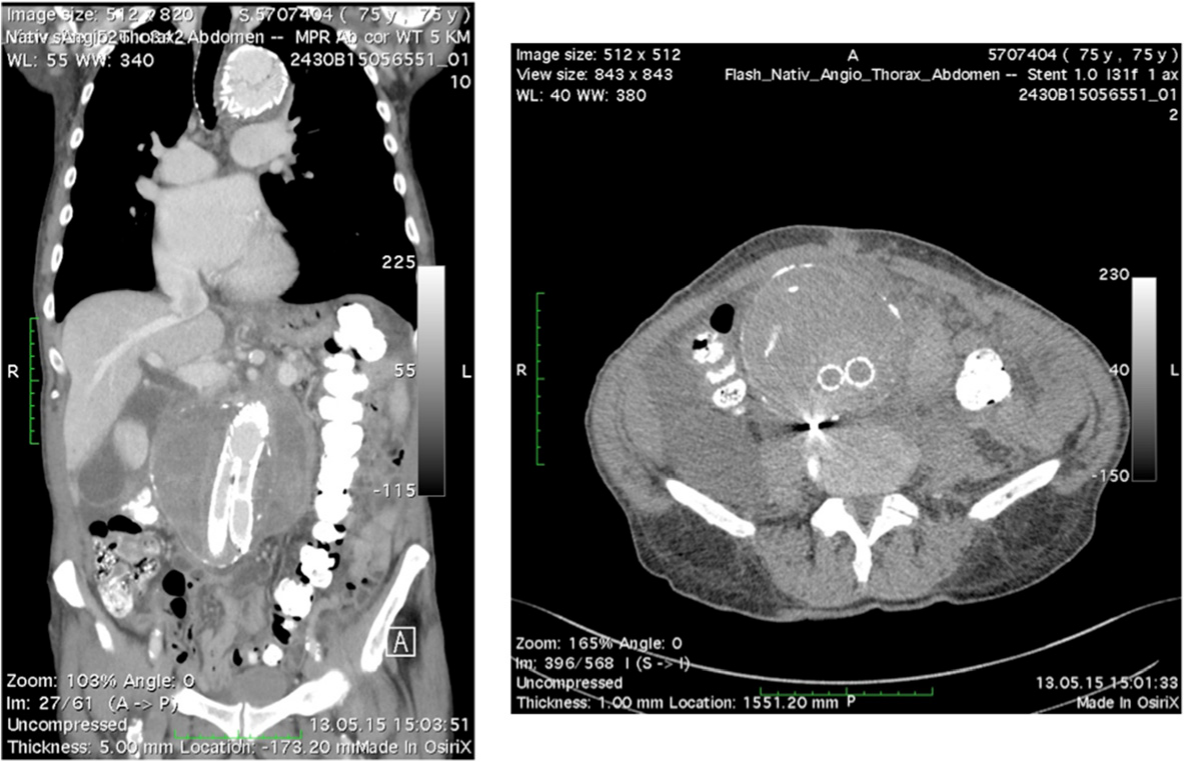
CT showing 10x9.5 cm infra-renal aortic aneurysm with a new para-aortic hematoma

**Fig. 2:**
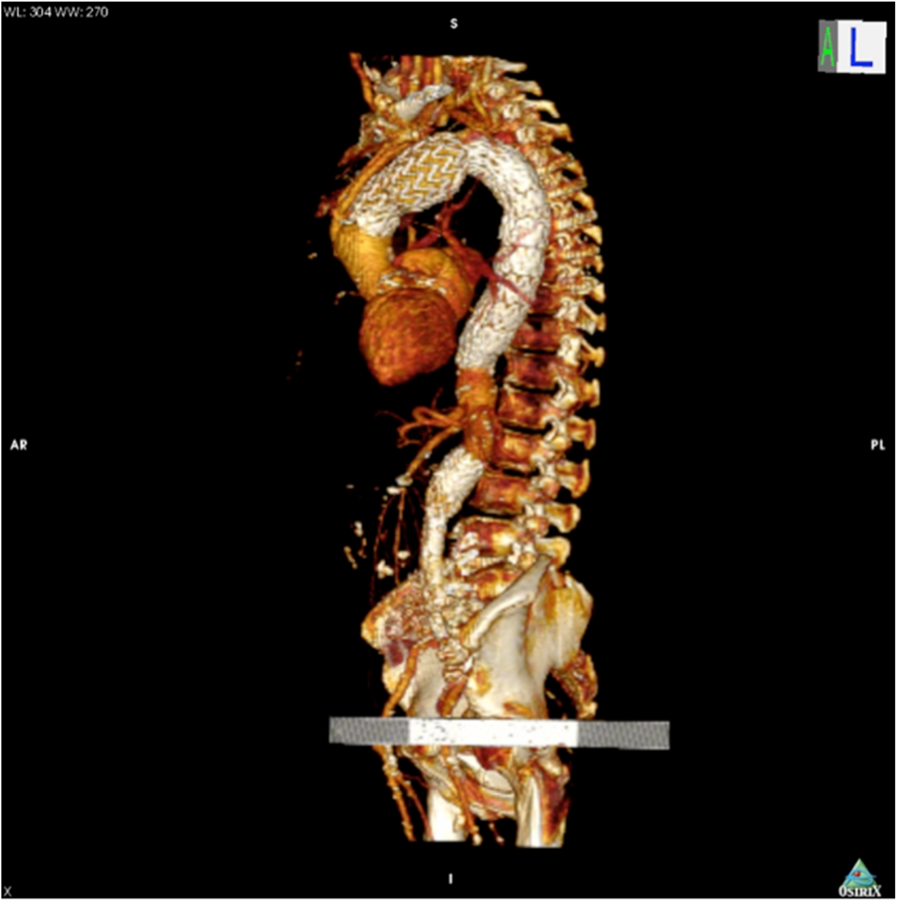
Sagittal CT angiogram three-dimensional (3D) reconstruction showing aortic arch and descending aortic stenting and aorto-bi-iliac prosthesis

A 75-year-old patient with a history of multiple endovascular and surgical interventions on the aorta was admitted to our clinic due to a rapid infra-renal aneurysm enlargement (10 cm × 9,5 cm) following endovascular aorto-bi-iliac stenting with a GORE Excluder stentgraft (W. L. Gore & Associates, Inc., Flagstaff, Arizona, USA) in September 2009 (Fig. 1). Furthermore, he was already treated with an endovascular stenting of an aneurysm in the descending thoracic aorta (2004) and an hybrid operation of a new developed aortic arch aneurysm with a combined endovascular stenting of the aortic arch (Gore TAG stentgraft (45 × 200mm)) and an aorto-truncal, aorto-subclavian and leftsided aorto-carotic bypass (Fig. 2).

Computed tomography (CT) showed an enlargement of the endovascularly excluded abdominal aortic aneurysm and a conspicuous new para-aortic hematoma and contrast agent enrichment in the aneurysm sac (Fig. 1). Preoperative transesophageal echocardiography revealed a critical aortic stenosis with a tri-leaflet heavily calcified valve, with an aortic valve area of 0.8 cm^2^ and a low-flow/low-gradient stenosis with a peak velocity of 2.3 m/s, a mean gradient of 11 mmHg and a severe reduced left ventricular function (LVEF 20 %).

After discussion in our multidisciplinary heart team we decided to perform a TF TAVI in combination with a simultaneous surgical abdominal aneurysm repair.

However, in a patient with previous aortic endovascular interventions, there is a risk for the balloon expandable Edwards Sapien valve frame to get stucked to the aortic stents. Hence, we used a special technique to facilitate easy device passage through the stents.

### Technique

Under general anesthesia TF TAVI procedure was performed in standard manner using a surgical cut-down to the ilio-femoral vessels and transcatheter aortic valve (Edwards SAPIEN 3 Transcatheter Heart Valve, 26 mm) was delivered using a 14 Fr Edwards eSheath Introducer Set (Edwards Lifesciences, Irvine, CA, USA). Balloon dilation of the stenotic aortic valve was performed with a 23 mm balloon, under rapid pacing using a temporary pacing.

**Fig. 3:**
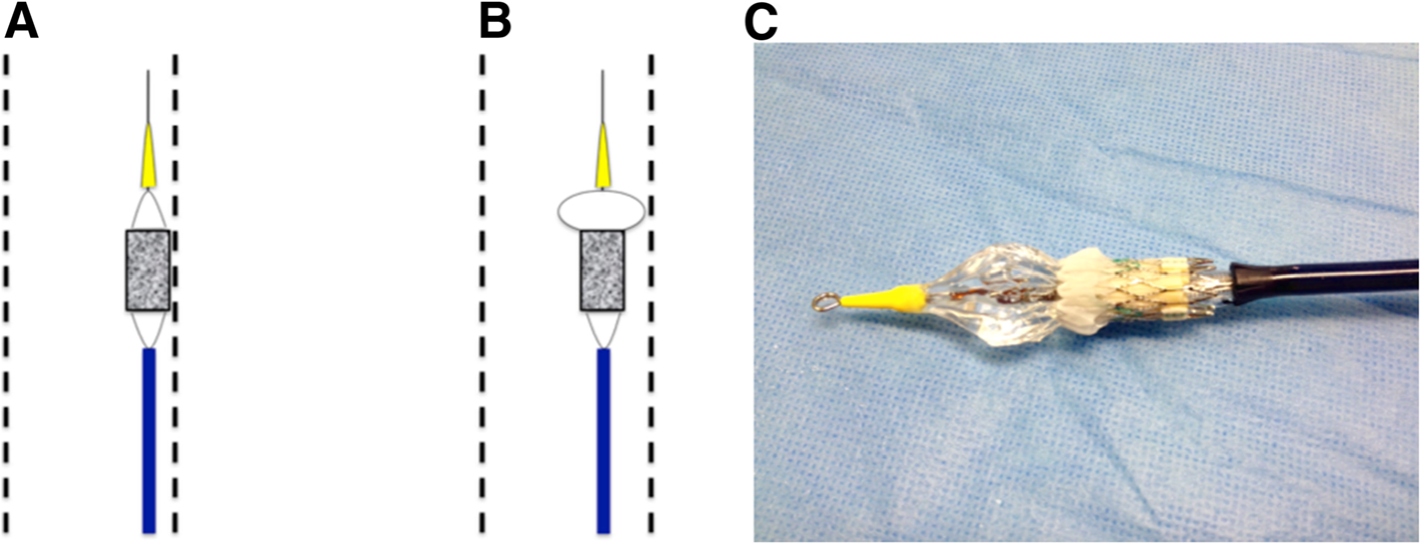
**a** Deployment of prosthesis in a patient with multiple endovascular stents. **b + c** Partial inflation of the distal balloon to minimize contact between valve frame and metal scaffold of endovascular stent

The critical part of the approach was the transition of the delivery system through the metal scaffold of the endovascular stents as it bears the risk that the uncovered Edwards valve frame gets stuck to the stent. Therefore we performed a partial inflation of the distal section of the balloon to minimize the contact between valve frame and endovascular stent and to allow an unimpaired passage of the valve (Fig. 3). This is achieved by injecting sufficient volume to inflate the balloon without flaring the TAVI device. The usual volume required is 2 to 3 ml. Once this is done the inflation device is locked. The whole system is then advanced with the distal balloon semi-inflated. After crossing the native valve and optimal placement, the inflation was continued and the SAPIEN 3 valve deployed in the usual manner.

Subsequent transesophageal echocardiography revealed an appropriate position of the implanted valve with an aortic valve area of 1.6 cm^2^ , no relevant gradient or paravalvular leakage.

Concomitant transperitoneal abdominal aortic aneurysm repair was then performed via median laparotomy. Inspection of the aneurysm offered no type I or type II endoleak. The aorto-bi-iliac stent was gently pulled out and infrarenal aortic reconstruction was performed by Dacron tube graft interposition.

Patients postoperative course was uneventful and he was discharged in stable condition after 8 days. A control CT demonstrated patent surgical anastomoses, endovascular grafts without endoleaks and echocardiography documented well-seated and well-functioning aortic valve prosthesis.

### Comment

In our case the presence of multiple endovascular stents aggravates the transfemoral approach due to the risk of getting the valve stuck to the metal scaffold of the stent. To achieve a distance between valve frame and endovascular stent we performed a partial inflation of the distal balloon and thus could pass the endovascular stents safely. This technique has been described by Davies et al. for eliminating balloon aortic valvuloplasty prior to transcatheter valve placement [3]. This has been shown to be effective in allowing accurate placement of a balloon-expandable device without the need for prior balloon aortic valvuloplasty.

In this case surgical infra-renal aortic aneurysm repair was chosen over endovascular aortic aneurysm repair (EVAR) due to the prior history of multiple interventions and the conspicuous preoperative CT suggesting the possibility of an endoleak and showing an additional para-aortic hematoma.

To our knowledge this is the first report of hybrid TAVI and surgical aortic aneurysm repair in a patient with prior multiple aortic interventions. There have been a few cases reporting on simultaneous TAVI and EVAR.

Drury-Smith et al. report on simultaneous TAVI in combination with an EVAR of an infra-renal aneurysm [4], whereas others describe a simultaneous approach with TAVI and EVAR of a thoracic or abdominal aneurysm [5, 6]. There have also been reports on sequential procedures in patients with aortic stenosis and aortic aneurysm performing TF [7] or transapical TAVI [8] followed by EVAR a few weeks later. Komlo et al. describe a special case, in which they combine transaortic TAVI and EVAR [9].

## Conclusion

We conclude that a partial inflation of the distal balloon in patients with TF TAVI and endovascular stenting minimizes the contact between valve frame and endovascular stent and leads to an easy passage of the valve through the stent. Hence the transfemoral approach in patients with former endovascular stenting can be performed successfully in a multidisciplinary approach.

## Consent

Written informed consent was obtained from the patient for publication of this Case report and any accompanying images. A copy of the written consent is available for review by the Editor-in-Chief of this journal.

## Abbreviations

CT: computed tomography
EVAR: endovascular aortic aneurysm repair
LVEF: left ventricular ejection fraction
TAVI: transcatheter aortic valve implantation
TF: transfemoral
